# Gut Microbiota Alteration with Moderate-to-Vigorous-Intensity Exercise in Middle School Female Football Athletes

**DOI:** 10.3390/biology14020211

**Published:** 2025-02-17

**Authors:** Jianlou Yang, Wei Zhang, Chen Dong

**Affiliations:** 1 School of Sport Management, Shandong Sport University, Jinan 250102, China; yangjianlou@sdpei.edu.cn; 2 School of Sports Leisure, Shandong Sport University, Jinan 250102, China; jlsvivi@163.com

**Keywords:** gut microbiota, exercise intensity, female football players

## Abstract

Background: The gut microbiota significantly influences health and metabolic processes. This study aimed to investigate the impact of exercise intensity on the gut microbiota of middle school female football athletes. Methods: In this four-week controlled comparative study, twenty-nine participants were divided into three groups: non-exercise group (NEG), moderate-intensity exercise group (MIEG), and vigorous-intensity exercise group (VIEG). They followed their respective exercise regimens for four weeks. Fecal samples were collected for 16S rRNA gene sequencing to evaluate microbiota composition. Results: The MIEG exhibited significantly greater microbial diversity compared to the NEG, while the VIEG showed lower diversity than the MIEG. Various microbiota profiles were identified, with the MIEG having higher levels of beneficial bacteria such as Bacteroides. Conclusions: Moderate-intensity exercise promotes a healthier gut microbiota compared to vigorous exercise in young female athletes. These findings underscore the potential of moderate exercise to enhance gut health and may inform training strategies for adolescent athletes.

## Simple Summary

This study explores how moderate-to-vigorous-intensity exercise affects the gut microbiota of middle school female football athletes. Gut microbiota is crucial for health, impacting metabolism and immunity. With increasing pressure on young athletes for both academic and sports performance, understanding the relationship between exercise intensity and gut health is essential. We compared three groups: those not exercising, those engaging in moderate-intensity exercise, and those engaging in vigorous-intensity exercise. Our findings showed that moderate exercise increased the diversity of gut bacteria, while vigorous exercise did not provide the same benefits. These results highlight the importance of exercise in supporting gut health, suggesting that moderate exercise may be more beneficial for maintaining a balanced microbiota in young female athletes. Understanding these dynamics can help coaches and parents aim for optimal training regimens that do not compromise health.

## 1. Introduction

The human intestinal tract is inhabited by various microbes consisting of bacteria, archaea, eukaryotes, and viruses. These microbes are referred to as the gut microbiota [1], composed of more than 3 million genes, collectively called the gut microbiome. In recent decades, there has been a growing interest in studying the impact of the microbiota on human health. This complex community of microorganisms plays a vital role in regulating various functions in the organism, such as metabolism and immunity [2]. One of the ways in which the microbiota contributes to metabolic functions is through the processing of indigestible dietary residues. As a result, they produce short-chain fatty acids (SCFAs) that contribute to the host’s metabolic homeostasis [3]. Furthermore, the microbiota also has a significant influence on the immune system. It stimulates the secretion of immunoglobulin (IgA) and the production of antimicrobial molecules. These molecules inhibit the proliferation and colonization of pathogenic bacteria, thereby promoting the development of gut-associated lymphatic tissue (GALT) and enhancing the host’s immune system [4]. Overall, the microbiota’s association with human health is a subject of growing interest. Its role in regulating metabolism and immunity through the production of SCFAs and stimulation of the immune system highlights its importance in maintaining overall well-being. Hence, the disruption of the balance of gut microbial communities can lead to physiological changes and increase the risk of metabolic disorders such as obesity, diabetes, cardiovascular disease, liver disease, as well as conditions related to gut health such as colorectal cancer and irritable bowel syndrome [5].

Exercise is well-known for its numerous health benefits, and researchers have recently turned their attention to exploring its connection with the gut microbiota [1]. Several studies have provided evidence that exercise can improve gut microbiome diversity [6,7,8,9]. During physical exercise, the cellular stress response depends on the intensity, with higher intensity being associated with an increased metabolic rate [10]. To improve and prevent metabolic disorders through regular exercise, the American College of Sports Medicine (ACSM) recommends moderate-intensity aerobic exercise for at least 30 min a day, five days a week, or a total of 150 min per week, or vigorous-intensity aerobic exercise for at least 20 min a day, three days a week [11].

Athletes, who undergo high-intensity training to enhance their physical performance, have been the focus of studies exploring the impact of exercise on the gut microbiota. Research has revealed that compared to sedentary individuals, athletes and physically active individuals generally display a greater diversity of fecal bacteria and an abundance of beneficial species [12,13,14]. Their gut microbiota also exhibits heightened microbial metabolism, particularly in carbohydrate and amino acid metabolic pathways [13,14,15]. Regular endurance exercise has been found to modulate the composition of the gut microbiota by reducing the presence of inflammation-associated proteobacteria [16]. In specific sports, such as competitive cycling, the relative abundance of certain bacterial species, like *Bacteroides* spp. and *Prevotella* spp., may be influenced by the volume of training [13]. Elite rugby athletes have been found to have a highly diverse gut microbiome, associated with upregulated pathways responsible for amino acid biosynthesis, carbohydrate metabolism, and short-chain fatty acid synthesis [17]. Similarly, college swimmer athletes have shown a positive correlation between the extent of training and gut microbiome involved in short-chain fatty acid synthesis [18]. Studies have also investigated the effects of light-to-moderate-intensity exercise training on changes in the microbiota profile in overweight adults [19], women [20], and obese children [21]. However, a short-term high-intensity interval training period was found to have no significant impact on the gut bacterial composition of lean and overweight men [22]. These findings provide support for the idea that physical exercise can induce alterations in the composition of the gut microbiota.

Despite extensive research on exercise and gut microbiota, there are several notable gaps in our current understanding. First, limited research is available on the gut microbiome composition, specifically in female middle school football athletes. Second, considering the academic pressure on middle school students, the optimal exercise intensity that can balance academic performance while maintaining athletic development and its effects on gut microbiome profiles remains unclear.

Therefore, this study had the following objectives: to investigate the impact of different exercise intensities (moderate vs. vigorous) on gut microbiota composition in female middle school football athletes; to compare the microbial diversity and abundance patterns among non-exercise, moderate-intensity exercise, and vigorous-intensity exercise groups; and to identify specific bacterial taxa that are significantly altered by different exercise intensities.

We hypothesized that both moderate and vigorous-intensity exercise would lead to significant alterations in gut microbiota composition compared to the non-exercise condition; moderate-intensity exercise would promote a more diverse and beneficial gut microbiota profile compared to vigorous-intensity exercise; and different exercise intensities would selectively influence the abundance of specific bacterial taxa associated with athletic performance and metabolism.

However, there is limited research available on the gut microbiome composition, specifically in female middle school football athletes. Therefore, further investigation into the gut microbiome of athletes, including female football players, is still an interesting topic of study. Additionally, considering the academic pressure on middle school students, the role of the intensity of physical activity that can ensure academic performance without compromising athletic performance in changing the gut microbiome profile has not been widely discussed. Therefore, the purpose of the current study was to examine the alterations in gut microbiota linked with moderate and vigorous-intensity exercise.

## 2. Materials and Methods

### 2.1. Design

This study recruited participants from Licheng No. 2 Middle School in Jinan through purposive sampling. The recruitment process involved distributing study information to all female football team members through their coaches. Written informed consent was obtained from both the participants and their legal guardians prior to study enrollment.

The inclusion criteria were as follows: (1) female football players aged 11–14 years; (2) regular members of the school football team; (3) willingness to maintain campus residence during the study period; (4) agreement to follow the prescribed exercise regimen; and (5) parental consent to participate. The exclusion criteria were as follows: (1) recent illness (within past month); (2) current use of antibiotics or probiotics; (3) known gastrointestinal disorders; (4) dietary restrictions or special dietary requirements; and (5) inability to complete the 4-week exercise program.

Twenty-nine participants who met all eligibility criteria were enrolled in the study. The sample size was primarily determined by practical considerations including the availability of eligible participants and resource constraints. While this is a pilot study with a limited sample size, it provides valuable preliminary data for future larger-scale investigations.

Twenty-nine healthy female football players from Licheng No. 2 Middle School in Jinan participated in this study. The study included a total of 29 athletes, with ages ranging from 11 to 14 years. The mean age of the female athletes was 13 ± 1.2 years, the mean height was 165 ± 4.6 cm, and the mean weight was 57.3 ± 13.8 kg. They were given the option to choose their desired group from the following: non-exercise group (NEG), moderate-intensity exercise group (MIEG), and vigorous-intensity exercise group (VIEG). Finally, 9 people were selected for the NEG, 11 people for the MIEG, and 9 people for the VIEG. To minimize variables affecting changes in gut microbiota other than exercise, 29 athletes stayed on campus and maintained a consistent daily routine during the trial period. They also chose to eat in the school cafeteria and maintained a similar diet and had no recent illnesses. Participants in the exercise group followed the ACSM exercise intensity guidelines for a period of 4 weeks. The exercise intensity of the MIEG was equivalent to 40–59% (at least 150 min of HRR per week). The VIEG engaged in high-intensity aerobic exercise with an intensity of ≥60% HRR, including 5 min warm-ups, at least 3 times a week, exceeding 30 min each time, and at least 90 min per week.

The exercise intervention protocol consisted of specific training programs for two exercise groups (MIEG and VIEG). Both groups participated in football-specific training, including technical drills (ball control, passing, shooting), tactical exercises, and small-sided games, supplemented with jogging, high-intensity interval running, strength training exercises, and dynamic stretching. The MIEG conducted 5 sessions per week, each lasting 30–40 min with intensity maintained at 40–59% Heart Rate Reserve (HRR), totaling a minimum of 150 min weekly. Each session included a 5 min warm-up, 20–30 min of main exercise, and a 5 min cool-down. The VIEG performed 3–4 sessions per week, each lasting 30–35 min at an intensity of ≥60% HRR, for a total of at least 90 min weekly. The sessions followed a similar structure, but the main exercise component lasted 20–25 min.

To ensure protocol adherence, comprehensive monitoring and control measures were implemented. All participants wore Polar H10 heart rate monitors during exercise sessions, with real-time data recorded and monitored by trained coaches who adjusted exercise intensity accordingly. Certified football coaches and two research assistants supervised all sessions, maintaining daily exercise logs and documenting attendance, duration, and intensity. The non-exercise group (NEG) was restricted to normal daily activities with a maximum walking duration of 15 min per session, monitored through activity trackers and daily logs. All participants, regardless of group assignment, followed standardized controls including dormitory residence, school cafeteria meals, regular sleep schedule (10:00 p.m.–6:00 a.m.), and daily routine monitoring by dormitory supervisors. The NEG participants engaged in seated leisure activities during physical education periods while maintaining regular academic schedules.

Prior to the study intervention, all participants were active members of the school football team with comparable training backgrounds, including three 90 min team practice sessions weekly, biweekly regional school league matches, and a total of 270 min of structured football training per week at a moderate to vigorous intensity (estimated 50–70% HRR). Their baseline physical activity profile showed 2.8 ± 0.9 years of football experience, 4.5 ± 0.5 weekly training hours, 1.9 ± 0.7 years of match play experience, twice-weekly physical education classes (90 min total), and daily physical activity averaging 7500 ± 1200 steps. To standardize the intervention, all regular team training was suspended during the 4-week study period, with participants following only their assigned group’s prescribed exercise protocols, thereby ensuring the better control of exercise variables and clearer assessment of intervention effects. All samples were collected in the morning, between 7:00 a.m. and 8:00 a.m., after an overnight fast, to ensure consistency across all participants.

The study was approved by the Ethics Review Committee of School of Sport Management, Shandong Sport University, with the approval number SD2024011.

### 2.2. Feces Sample Collection and DNA Extraction

The implementation of group-specific exercise protocols, daily monitoring of exercise compliance, weekly activity log reviews, and standardized diet maintenance during Weeks 1–4 (intervention phase) concluded with the final fecal sample collection (48 h after last exercise session) and post-intervention data collection in Week 4 (post-intervention phase). Fecal samples were collected in a sterilized 50 mL tube without additives, ethanol, or stabilizing solutions, immediately stored and placed on frozen packages, and delivered to the laboratory to be stored at −80 °C within two hours. The sample was removed from the refrigerator and an appropriate amount (between 0.2 and 0.5 g) was swiftly taken out, added to a centrifuge tube containing the extraction lysate. Subsequently, the sample was ground using the Tissuelyser-48 at 60 Hz. Total genomic DNA samples were extracted using the OMEGA Soil DNA Kit (M5635-02) (Omega Bio-Tek, Norcross, GA, USA), following the manufacturer’s instructions, and stored at −20 °C prior to further analysis. The quantity and quality of extracted DNA were measured using a NanoDrop NC2000 spectrophotometer (Thermo Fisher Scientific, Waltham, MA, USA) and agarose gel electrophoresis, respectively.

### 2.3. 16S rRNA Gene Amplicon Sequencing

PCR amplification of the bacterial 16S rRNA genes V3–V4 region was performed using the forward primer 338F (5′-ACTCCTACGGGAGGCAGCA-3′) and the reverse primer 806R (5′-GGACTACHVGGGTWTCTAAT-3′). Sample-specific 7-bp barcodes were incorporated into the primers for multiplex sequencing. The PCR components contained 5 μL of buffer (5×), 0.25 μL of Fast pfu DNA Polymerase (5 U/μL), 2 μL (2.5 mM) of dNTPs, 1 μL (10 μM) of each Forward and Reverse primer, 1 μL of DNA Template, and 14.75 μL of ddH_2_O. Thermal cycling consisted of initial denaturation at 98 °C for 5 min, followed by 25 cycles consisting of denaturation at 98 °C for 30 s, annealing at 53 °C for 30 s, and extension at 72 °C for 45 s, with a final extension of 5 min at 72 °C. PCR amplicons were purified with Vazyme VAHTSTM DNA Clean Beads (Vazyme, Nanjing, China) and quantified using the Quant-iT PicoGreen dsDNA Assay Kit (Invitrogen, Carlsbad, CA, USA). After the individual quantification step, amplicons were pooled in equal amounts, and pair-end 2 × 250 bp sequencing was performed using the Illlumina NovaSeq platform with NovaSeq 6000 SP Reagent Kit (500 cycles) at Shanghai Personal Biotechnology Co., Ltd. (Shanghai, China).

### 2.4. Sequence Analysis

Microbiome bioinformatics were performed using QIIME2 2019.4 [23], with slight modifications according to the official tutorials “https://docs.qiime2.org/2019.4/tutorials/ (accessed on 7 June 2024)”. In brief, the raw sequence data were processed through several steps. First, the demux plugin was used to demultiplex the data, followed by primer cutting using the cutadapt plugin [24]. Next, the sequences underwent quality filtering, de-noising, merging, and chimera removal using the DADA2 plugin [25]. To construct a phylogeny, non-singleton amplicon sequence variants (ASVs) were aligned using mafft [26], and a phylogeny was built using fasttree2 [27]. Various alpha diversity metrics, including Chao1, Observed species, and Shannon metrics [28,29,30,31,32,33,34,35], were estimated using the diversity plugin. To ensure consistency, samples were rarefied to 20,000 sequences per sample. Taxonomy was assigned to the ASVs using the classify-sklearn naïve Bayes (2024.10.0) taxonomy classifier in the feature-classifier plugin [36], with the SILVA Release 132 Database [37] serving as the reference database.

### 2.5. Bioinformatics and Statistical Analysis

The sequence data analysis was primarily conducted using QIIME2 and R packages (v3.2.0). Alpha diversity indices at the ASV level, such as Chao1 richness estimator, observed species, and Shannon diversity index, were calculated using the ASV table in QIIME2. These indices were further visualized as box plots. ASV-level ranked abundance curves were generated to compare the richness and evenness of ASVs across samples. For all statistical analyses, the significance level was set to *p* < 0.05 or the listed *p* value. R was used for all statistical analysis and visualization (http://www.R-project.org/, accessed on 7 June 2024). Benjamini Hochberg corrected *p* values were based on Wilcoxon rank test or Kruskal–Wallis test (significance threshold *p*  <  0.05).

For beta diversity analysis, the ASV tables were used to generate a matrix, and constrained principal coordinate analysis (CPCoA) was performed and displayed by ggplot2 package in R (Version 3.5.2). Bray–Curtis metrics were used to investigate the structural variation in the microbial communities among samples [38,39]. The significance of differentiation in microbiota structure among groups was assessed using PERMANOVA (Permutational multivariate analysis of variance) in QIIME2 [40,41,42,43,44].

Taxonomic composition was investigated at the phylum and genus levels [45,46,47,48,49,50]. Random forest analysis was conducted in QIIME2 with default settings to discriminate samples from different groups [51]. Nested stratified k-fold cross-validation was used for automated hyperparameter optimization and sample prediction with k-fold cross-validations set to 5.

Co-occurrence network analysis was performed using SparCC (0.1.0-0) analysis. The pseudocount value in SparCC was set to 10^−6^, and the cutoff of correlation coefficients was determined as 70 using random matrix theory-based methods implemented in the R (4.4.2) package “RMThreshold”. Based on the correlation coefficients, a co-occurrence network was constructed with ASVs represented as nodes and edges representing correlations between them. The network was visualized using the R packages “igraph” and “ggraph”.

Microbial functions were predicted using PICRUSt2 (Phylogenetic investigation of communities by reconstruction of unobserved states) [52] based on the MetaCyc (https://metacyc.org/, accessed on 7 June 2024) and KEGG (https://www.kegg.jp/, accessed on 7 June 2024) databases. We also utilized the MICOM framework to construct a metabolic flux model focusing on acetate, propionate, and butyrate production. The model parameters were selected based on the microbial gene abundances obtained from our previous analyses, specifically targeting carbohydrate fermentation pathways. This approach allows us to predict the SCFA fluxes associated with specific microbial taxa.

## 3. Results

### 3.1. Sequencing Analysis and Data Processing

A total of 2,837,944 raw sequences were generated. Of these, 1,520,945 high-quality reads were retained after denoising and removing low-quality and chimeric sequences using DADA2, subsequently generating 35,783 ASVs. Among them, 808 ASVs, representing 1.54% (23,432 sequences) of the total high-quality sequences, could not be identified to any known phylum based on the Greengenes database (Release 13.8, https://ftp.microbio.me/greengenes_release/2022.10/, accessed on 7 June 2024). Illumina-based reads from the lab and field controls showed negligible signals of DNA contamination and hence were not included in the subsequent analyses.

### 3.2. Effects of Different Intensity Exercise on the Gut Microbiome Diversity

**Figure 1:**
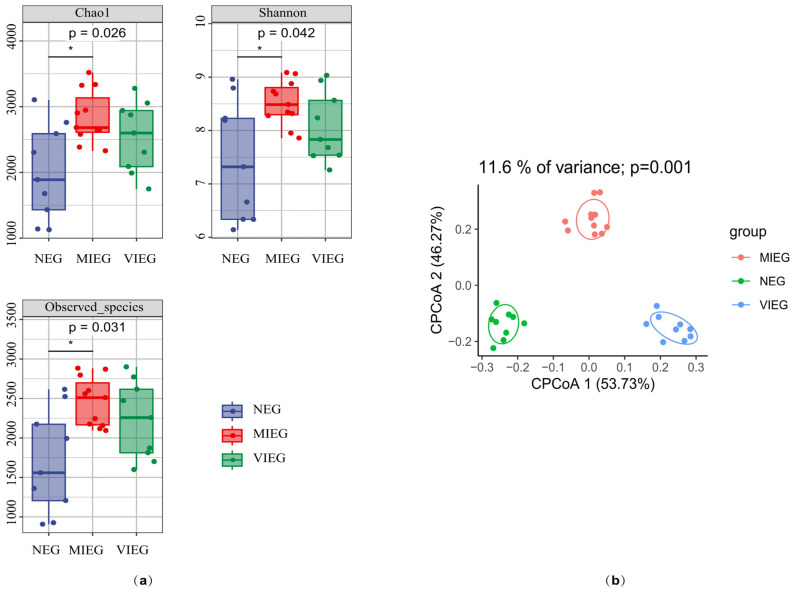
Gut microbiome profiles were analyzed across different exercise intensities. (**a**) Alpha diversity estimates of the bacteria communities were compared using Kruskal–Wallis test (*p* < 0.05). (**b**) Cluster-based principal coordinate analysis (CPCoA) was conducted at the ASV level using Bray–Curtis distance to compare the data between groups. The groups included the non-exercise group (NEG), moderate-intensity exercise group (MIEG), and vigorous-intensity exercise group (VIEG). * *p*  ≤ 0.05.

The alpha diversity of the gut microbiome was assessed using various indices, including Chao1, Shannon, and Observed_species (number of observed ASVs). All these metrics consistently showed that the MIEG (Group A) exhibited significantly greater diversity in the bacteria community compared to the NEG (Group B) (Chao1: *p* = 0.026, Shannon: *p* = 0.042, Observed_species: *p* = 0.031; Figure 1a. As the intensity of exercise increased, the diversity increased. However, during high-intensity exercise, the diversity was actually lower than during moderate-intensity exercise.

We also examined the composition of bacteria beta diversity among the different groups. The cluster-based principal coordinate analysis (CPCoA) showed significant dissimilarity (*p* = 0.001, assessed using *PERMANOVA*) at the ASV level, based on the Bray–Curtis distance. CPCoA revealed distinct clustering patterns and explained 11.6% of the total variation in bacteria communities across different exercise intensities. The Bray–Curtis dissimilarity metrics indicated that CPCoA1 contributed to 53.73% of the total variation, while CPCoA2 contributed to 46.27% of the total variation. The bacterial communities of the NEG group were clearly distinguishable from those of the MIEG and VIEG groups. We had used the stat_ellipse (level = 0.68) function from the ggplot2 package, which corresponds to a 68% confidence interval for normally distributed data (Figure 1b).

### 3.3. Effects of Different Intensity Exercise on the Community Composition of Gut Microbiome

**Figure 2:**
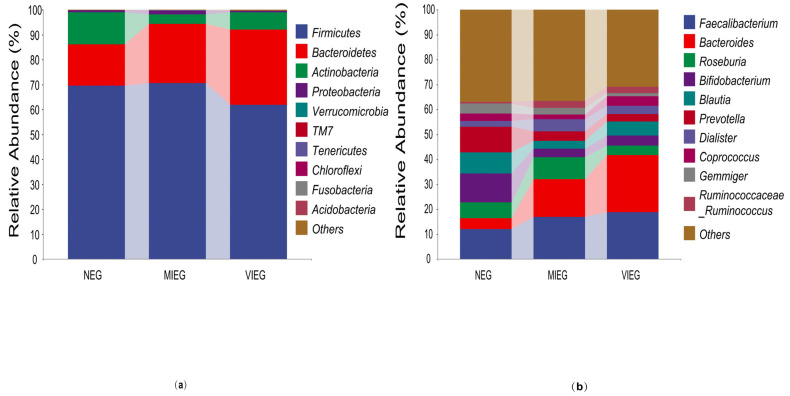
Gut microbiome community composition in different exercise intensity samples is represented by the relative sequence abundance of bacteria phyla (**a**) and the relative abundance (%) of major genera (**b**).

Ten phyla, including *Firmicutes*, *Bacteroidetes*, *Actinobacteria*, *Proteobacteria*, *Verrucomicrobia*, *TM7*, *Tenericutes*, *Chloroflexi*, *Fusobacteria*, and *Acidobacteria*, were identified using the Greengenes reference (13.8) database for all samples. Unclassified reads in the bacteria phylum were removed from the sequencing data. In Figure 2a, it can be observed that the bacteria in the non-exercise group (NEG) primarily consist of *Firmicutes* (69.42%), *Bacteroidetes* (16.72%), and *Actinobacteria* (12.85%). In contrast, the moderate-intensity exercise group (MIEG) and vigorous-intensity exercise group (VIEG) were predominantly composed of *Firmicutes* (70.54% and 61.82%, respectively), *Bacteroidetes* (23.78% and 30.09%, respectively), and *Actinobacteria* (3.88% and 7.07%, respectively) at the phylum level.

The groups of samples displayed varying proportions of taxonomic compositions at the genus level. *Bacteroides* was found to be more prevalent in the moderate-intensity exercise group (MIEG) and vigorous-intensity exercise group (VIEG), accounting for 15.25% and 22.79% of the genera, respectively. Conversely, its abundance in the non-exercise group (NEG) was relatively low, at 4.33%. Additionally, the percentages of *Prevotella* and *Bifidobacterium* in the NEG were higher compared to the MIEG and VIEG (10.35% and 11.51% in NEG, compared to 3.80%, 3.18%, 3.07%, and 3.94% in MIEG and VIEG, respectively; Figure 2b).

### 3.4. Taxonomic Biomarkers Analysis of Gut Microbiota Communities During Different Intensities of Exercise

**Figure 3:**
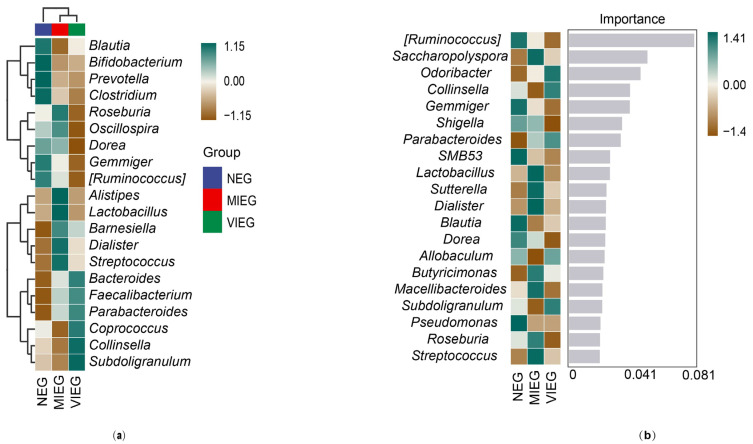
To analyze the taxonomic biomarkers of gut microbiota communities during different intensities of exercise, two approaches were employed. (**a**) Heatmap was generated to visualize the relative abundance of the top 20 intensity-related biomarkers. (**b**) Random forest approach was utilized to identify and rank the 20 genera associated with different intensities based on their contribution, from largest to smallest.

To explore the differences in microbial community composition (beta diversity) primarily attributed to species distribution, two approaches were employed. Firstly, a heatmap was used to compare the variations in species composition between groups and analyze the distribution trends of species abundance within each group. The heatmap was based on the abundance data of the top 20 genera with average abundance (Figure 3a). Secondly, the random forest algorithm was utilized to identify potential taxonomic biomarkers and classify microbial community samples effectively, robustly, and accurately (Figure 3b).

At the genus level, we performed a tenfold cross-validation to obtain the importance score of species for the classifier model. From top to bottom, the importance of species to the model decreases progressively; these highly important species can be considered as marker species for inter group differences. The importance scores of *Ruminococcus*, *Saccharopolyspora*, and *Odoribacter* were relatively high, at 0.081, 0.051, and 0.047, respectively.

### 3.5. Co-Occurrence Network of Gut Microbiota

Since microorganisms can compete or synergize with each other, the interactions between different microbial strains were one of the main drivers of changes in population structure. We used network inference analysis based on the relationships between microbial members to search for inherent patterns of co-occurrence or co-exclusion in gut microbial communities.

**Figure 4:**
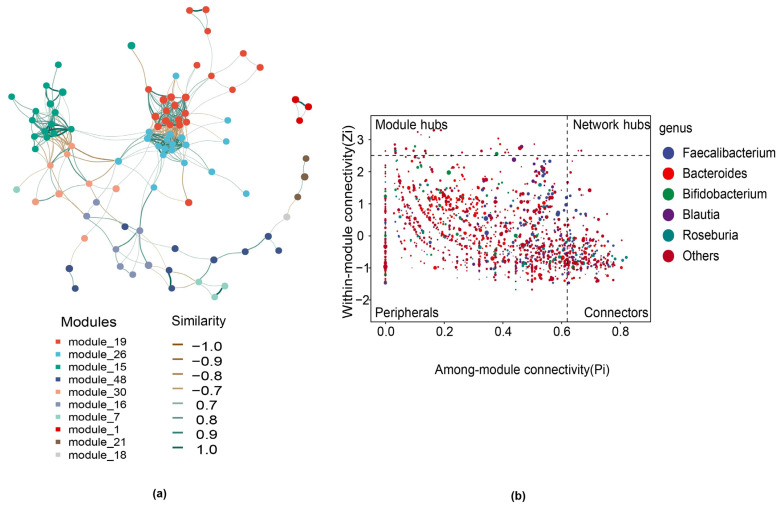
Correlation network analysis of the bacteria community. (**a**) Species from different phyla are represented by nodes in the network diagram. Bacteria at different phylum levels are distinguished by color. The size of each node corresponds to the quantity of the corresponding bacteria. The connection between any two nodes indicates two genera with a markedly high correlation (Spearman’s |r| > 0.7; *p* < 0.01). (**b**) The nodes (ASV/OTU) in the network can be divided into four parts using Zi and Pi values, namely peripherals, connectors, module hubs, and network hubs.

The network diagram showed the degree of correlation between gut microbiota communities (Figure 4). Nodes represent the ASVs in the sample, and their size was proportional to their abundance (measured in log2 (CPM/n)). The top 10 modules with the most nodes were identified by different colors. The edge between nodes indicated the existence of correlation between two connected nodes.

The network diagram showed a strong positive correlation between *Ruminococcaceae* and *Christensenellaceae* (Spearman’s r = 0.88; *p* < 0.01) and *Butyricicoccus* and *Rikenellaceae* (Spearman’s r = 0.85; *p* < 0.01) (Figure 4a). Zi and Pi score values were calculated for each node in the current co-occurrence network, based on the modular segmentation results. The Zi value represents the within-module connectivity of each node, while the Pi value represents the connectivity among modules. The role of each node in the network was determined based on their Zi and Pi score values. Through analysis, the species identified at the genus level are *Faecalibacterium*, *Bacteroides*, *Bifidobacterium*, *Blautia*, and *Roseburia* (Figure 4b).

### 3.6. PICRUSt2 Analysis

**Figure 5:**
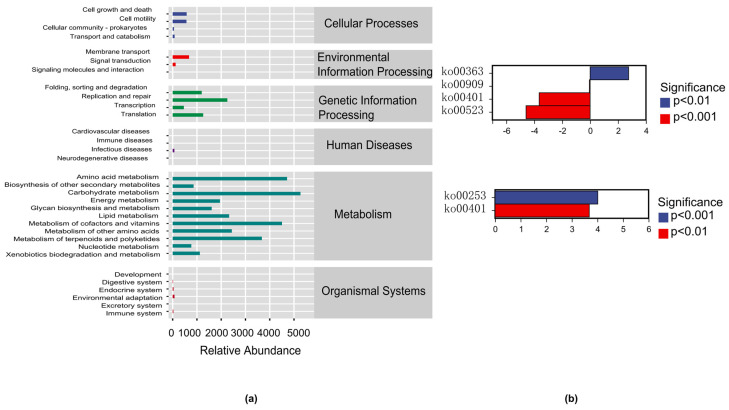
The results of PICRUSt2 function prediction analysis. (**a**) Relative abundances of bacterial predicted functional (KEGG) pathways. (**b**) Analysis of differences in gut microbiota metabolic pathways under different exercise intensities.

Microbial ecology research also focuses on assessing the functional potential of microbial communities. The core of the KEGG database is the Biological Metabolic Pathway Analysis Database (KEGG Pathway Database), (http://www.genome.jp/kegg/pathway.html, accessed on 7 June 2024). Among them, metabolic pathways were classified into six categories, including metabolism, genetic information processing, environmental information processing, cellular processes, organic systems, and human diseases. Each type of metabolic pathway was further divided into multiple levels. At present, the second level included a total of 45 metabolic pathway subfunctions, the third level corresponds to the metabolic pathway map, and the fourth level corresponds to various KO groups (KEGG orthologous groups) on the metabolic pathway (Figure 5).

A normalized pathway/group abundance table was used to calculate the average abundance or total number of second level pathways/classifications based on different exercise intensities. The normalization process involved scaling the observed abundances by a normalization factor to eliminate biases resulting from unequal sampling efforts. This approach ensures that the abundance values are comparable across different samples and conditions. Specifically, the abundance of each pathway or classification was normalized to the total number of reads in each sample, resulting in relative abundance values. This method allows us to accurately compare the abundance of pathways and classifications across different exercise intensities. The results showed that metabolism and genetic information processing were the dominant functions among the six categories. At the second level, the relative abundance of carbohydrate metabolism, amino acid metabolism, and metabolism of cofactors and vitamins were the highest (Figure 5a).

The analysis results of PICRUSt2 indicated significant differences in ko00363 (Bisphenol degradation), ko00401 (Neobiotin biosynthesis), and ko00523 (Polyketose unit biosynthesis) between the NEG and MIEG. Moreover, we founded no significant difference in metabolic pathways between the NEG and VIEG, while there were significant differences in ko00253 (Tetracycline biosynthesis) and ko00401 (Novobiocin biosynthesis) between the MIEG and VIEG (Figure 5b).

Our analysis identified several candidate microbes significantly associated with predicted SCFA fluxes. These included *Bacteroides* and *Roseburia*, which may play a key role in the observed shifts in the SCFA production capacity within our cohort.

## 4. Discussion

Our investigation into the impact of varying exercise intensities on the gut microbiome of high school female football players reveals significant insights into the complex interactions between physical activity and microbial communities. The findings illustrate how different intensities of exercise can variably influence microbial diversity and composition, which may have broader implications for athlete health and performance.

Our analysis revealed that moderate-intensity exercise significantly enhanced the alpha diversity of the gut microbiota compared to the non-exercise group. This aligns with previous studies that have established a correlation between increased gut microbial diversity and various health benefits, including improved metabolic functions and immune responses [6,7,8]. Interestingly, while we observed an increase in diversity with moderate-intensity exercise, the alpha diversity was lower during high-intensity exercise. This paradoxical finding may suggest that high-intensity training could induce a state of stress on the microbiome, affecting its composition and functionality. Future research should investigate the specific stress responses triggered by high-intensity exercise and their consequent effects on microbiota resilience and recovery.

Comparative analyses of the gut microbiota composition at the phylum and genus levels revealed that different exercise intensities were associated with varying microbial proportions. The dominance of *Firmicutes* in both exercise groups mirrors findings that link these bacteria to beneficial metabolic profiles, including short-chain fatty acid (SCFA) production [3,4]. Furthermore, the increased relative abundance of *Bacteroides* in the exercise groups signifies an adaptation that is likely associated with metabolic enhancement, as these genera are known for their roles in carbohydrate metabolism and energy harvest [17].

Conversely, the reduced abundance of *Prevotella* and *Bifidobacterium* in the exercise groups emphasizes the dynamic nature of the microbiota influenced by physical activity. While *Bifidobacterium* is often associated with health benefits, its decreased presence in high-intensity exercise could reflect a competitive interplay among microbial groups under physiological stress conditions encountered during intense training [16].

The random forest analysis identified several genera, including *Ruminococcus* and Saccharopolyspora, as crucial taxonomic markers for differentiating among the exercise intensity groups. The importance of *Ruminococcus* suggests its pivotal role in SCFA production, reaffirming the potential for exercise-induced alterations to promote metabolic health. Conversely, species like Saccharopolyspora, while less understood, may offer new avenues for research into the functional contributions of less prevalent microbes in athletic populations.

The co-occurrence network analysis elucidated the interactions among different microbial taxa, revealing that exercise intensity modulates microbial connectivity. The robust positive correlations observed among key genera, such as *Ruminococcaceae* and *Christensenellaceae*, indicate that certain microbial communities thrive synergistically under specific exercise regimes. Understanding these intricate relationships could pave the way for microbial interventions aimed at optimizing gut health and enhancing athletic performance.

Through PICRUSt2 analysis, we identified significant alterations in predicted metabolic pathways associated with exercise intensity, particularly enhancing carbohydrate and amino acid metabolism pathways in the moderate-intensity group. These pathways are crucial for athletic performance, as they directly impact energy metabolism and muscle recovery [53]. Carbohydrate metabolism is the primary energy source for muscle contraction, especially during high-intensity exercise, and its efficiency is vital for sustaining physical activity. Amino acid metabolism, on the other hand, is essential for muscle protein synthesis and repair, which are critical for post-exercise recovery and adaptation [54]. The observed functional shifts with increased microbial diversity suggest a potential link between microbial metabolism and host athletic performance [55]. Specifically, a more diverse and metabolically active gut microbiota can enhance the host’s ability to utilize nutrients, reduce inflammation, and improve muscle recovery, all of which are critical for optimal athletic performance. The absence of significant differences in certain metabolic pathways between the moderate and vigorous exercise groups may indicate a threshold effect or saturation point in microbial adaptations to physical stress [56], highlighting the complexity of microbiome responses to varying exercise intensities.

Our PICRUSt2 analysis has enabled us to predict metabolic pathways linked to exercise intensity, and we have extended this to include a metabolic flux model for acetate, propionate, and butyrate using the MICOM (61.0) approach. This model highlights the candidate microbes that may be responsible for the predicted SCFA fluxes observed in our study. By leveraging the microbial gene abundances for carbohydrate fermentation pathways already assessed, we have been able to provide a more comprehensive view of the SCFA production capacity in the gut. Our analysis reveals that the production of SCFAs, particularly butyrate and propionate, is influenced by specific microbial taxa within our cohort. These taxa, including *Bacteroides* and *Roseburia*, are known to be involved in SCFA production and have been previously associated with changes in response to exercise. The functional redundancy seen in various bacteria in the gut suggests that a diverse microbiome may offer greater stability and adaptability in the face of disturbances, such as changes in exercise intensity. Our findings underscore the importance of considering the microbiome in exercise physiology, as it relates to energy metabolism and muscle recovery.

Our study contributes to the growing body of literature elucidating the relationship between exercise intensity and gut microbiome dynamics. The findings underscore the need for tailored exercise regimens that consider their impact on gut health, particularly for young athletes balancing academic and athletic activities. Future research should investigate the long-term implications of these microbial changes and explore potential interventions, such as dietary modifications or probiotic supplementation, to optimize gut health and overall performance in athletes. Furthermore, conducting studies that encompass a diverse range of populations and various exercise modalities has the potential to provide deeper insights into the intricate relationship between physical activity and the gut microbiota. One of the primary limitations of this study is the small sample size, with only 29 athletes across three groups. Small sample sizes can limit the generalizability of our findings and may affect the statistical power of our analysis. This constraint could potentially introduce bias and reduce the ability to detect significant differences among groups. Given the exploratory nature of our study and the complexity of the microbiome, larger sample sizes would be required to confirm our findings and to establish more definitive conclusions. The small sample size also restricts our ability to account for individual variability in microbiome composition and its response to exercise intensity. Future studies with larger cohorts would enable more robust statistical analyses and could provide a more comprehensive understanding of the relationship between exercise intensity, microbiome composition, and SCFA production. Despite these limitations, we believe our study provides valuable preliminary insights into the impact of exercise intensity on the gut microbiome among middle school athletes. The findings from this study can serve as a foundation for future research with larger sample sizes to further explore these relationships.

## 5. Conclusions

This study illustrates the significant influence of exercise intensity on the gut microbiome composition and diversity among middle school female football players. Moderate-intensity exercise proved to be particularly beneficial for enhancing microbial diversity, while high-intensity training presented unique challenges that led to shifts in gut microbial richness. The identification of specific bacterial taxa and metabolic pathways associated with varying intensities underscores the possibility of elaborate personalized training and nutritional approaches to cultivate a healthier gut microbiome in athletes.

Future research is essential to establish causal relationships between exercise intensity, gut microbiome dynamics, and their collective impact on health and performance. Understanding these interactions can pave the way for targeted interventions, potentially leveraging the gut microbiome to enhance athletic performance and overall well-being. Continued exploration into this area will not only benefit athletes but also contribute valuable knowledge applicable to broader populations facing sedentary lifestyles and associated health risks.

## Data Availability

The data presented in this study are original and were collected specifically for this research. They are not openly available in a public repository but are available from the corresponding author upon reasonable request and with the necessary considerations for participant confidentiality and ethical restrictions.
